# Correlation of serum complement factor 5a level with inflammatory response and cognitive function in patients with Alzheimer’s disease of different severity

**DOI:** 10.1186/s12883-023-03256-w

**Published:** 2023-09-07

**Authors:** Zhilian Li, Huifang Wu, Yi Luo, Xianpei Tan

**Affiliations:** 1 Department of Neurology, The First People´s Hospital of Jingzhou City, No.8 HangKong Road, Shashi District, 434100 Jingzhou City, Hubei Province P.R. China; 2 Yangtze University, 434023 Jingzhou City, Hubei Province P.R. China

**Keywords:** Alzheimer’s disease, Complement factor 5a, Inflammatory response, Cognitive function, Pearson test, Logistic multivariate regression, Receiver operating characteristic curve, Kaplan-Meier

## Abstract

**Background:**

Alzheimer’s disease (AD) is a common cause of dementia. Serum complement factor 5a (C5a) is exceedingly implicated in AD. We explored the role of C5a levels in AD patients of different severity.

**Methods:**

Mild, moderate, and severe AD patients, and healthy controls were included. C5a and pro-inflammatory factor (TNF-α, IL-1β, IL-6, CRP) levels were assessed by ELISA, and cognitive function was evaluated by Mini-Mental state examination (MMSE) score. The correlations between C5a, inflammatory factor levels, MMSE score, and plasma Aβ42/Aβ40 ratio were analyzed by Pearson tests. Independent risk factors for AD aggravation were assessed by logistic multivariate regression analysis. According to the cut-off value of receiver operating characteristic (ROC) curve analysis of C5a level, AD patients were assigned into low/high expression groups, and severe AD incidence was compared. Severe AD cumulative incidence was analyzed by Kaplan-Meier curve.

**Results:**

Serum C5a, TNF-α, IL-1β, IL-6 and CRP levels were raised, and MMSE score was lowered in AD. Serum C5a, TNF-α, IL-1β, IL-6 and CRP levels in severe AD patients were higher than those in mild/moderate AD patients, but there were no significant differences in these cytokines between moderate and mild AD groups. The MMSE score of severe AD patients was lower than that of mild/moderate AD patients. Serum C5a level was positively correlated with serum TNF-α, IL-1β, IL-6, and CRP levels, and negatively correlated with MMSE score, with no obvious correlation with plasma Aβ42/Aβ40 ratio. Serum C5a level was one of the independent risk factors for AD aggravation. The occurrence of severe AD might be related to an increase in serum C5a level.

**Conclusion:**

Serum C5a level increased with AD severity, and its expression was positively correlated with serum pro-inflammatory factor levels, and negatively correlated with cognitive function.

## Introduction

Alzheimer’s disease (AD) is a complex neurodegenerative disease, which is characterized by neuron progressive loss, typically causing severe impairment in cognitive functions of patients including memory and learning [1]. Nowadays, as the most prevalent cause of dementia, AD is a growing health concern across the globe, with huge implications for society and individuals [2]. The majority of AD is multifactorial with complex interactions across polygenic risk factors (especially apolipoprotein E), lifestyle, and environmental factors [3]. Neurodegeneration (such as neuronal loss and/or atrophy) can be attributed to intraneuronal neurofibrillary tangles consisting of hyperphosphorylated tau, extraneuronal toxic amyloid proteins and oligomers, region-specific repressed cerebral glucose metabolism, mitochondrial dysfunction, and synaptic dysfunction [4]. The 2 central pathological hallmarks of AD are neurofibrillary tangles and amyloid plaques, and the deposition of amyloid β (Aβ) triggers dysfunction of neurons and death in the brain [5]. The 3 identified (classic) biomarkers in cerebrospinal fluid (CSF) are Aβ42, tau protein phosphorylated at a threonine residue at position 181 (τP-181), and total tau protein (τT), which are the gold standards for liquid based diagnosis of AD [6, 7]. Through a better understanding of AD inflammation, it is possible to develop anti-inflammatory approaches, which are likely help to slow the progression of this devastating disease [8]. Therefore, it is pivotal to study the mechanism of AD through the aspect of inflammation and find efficient targets for AD alleviation.

Blood based biomarkers as a possible diagnostic aid for CSF biomarker have recently received widespread attention [9]. Blood sampling is an easy, non-invasive, and acceptable procedure without complications, requiring no hospitalization, and can be performed in outpatient wards or communities, which allows for the collection of larger sample sizes and is suitable for a wider range of analyte measurements [10]. The complement system is an extremely pivotal component of the innate immune system with the ability to identify and clear infectious challenges that invade the central nervous system through damaged blood-brain barriers, which has an essential property in the defense of the host to ultimately maintain the homeostasis of the brain, and multiple complement proteins have been identified to be co-localize with amyloid plaques in AD patients, indicating a role of the in AD [11, 12]. The activation of the complement system plays an important role in AD neuroinflammatory injury, and the complement mainly exists in serum and tissue fluid, and activated complement can have enzymatic activity [13]. A previous study has recognized the involvement of complement cascade in the pathogenesis of AD, with high levels of complement produced in the affected areas of the AD brains, and the complete activation of the classical complement pathway may have a significant impact on the pathology of AD [14]. Complement proteins and many inflammatory mediators are locally produced by brain cells [15]. Complement activation in the central nervous system is a double-edged sword. Brain complement activation may promote tissue damage by inducing neuronal cell death (allergic toxins and membrane attacks) and promote the release of pro-inflammatory cytokines by activated glial cells. On the other hand, complement activation can have beneficial effects on the brains by promoting neuroprotection and tissue remodeling [16]. Excessive downstream complement activation leads to the generation of C5a, and C5a engages with its receptor C5aR1 to trigger inflammation, injury, and neuronal death [17]. C5a induces neuronal cell apoptosis, while C5a/C5aR1 signaling increases vascular permeability and the production of inflammatory cytokines [18]. Complement protein C5a promotes the β-amyloid-mediated neuroinflammatory response in microglia in AD [19]. The life of C5a is brief, and it rapidly transforms into C5a-des Arg in the body [20]. C5a receptors (C5aR1) is the pro-inflammatory receptor for C5a, and the C5aR1 antagonist beneficial effects in AD mice may be due to neuron protection from the toxic effects of C5a [21]. The apoptosis pathway is associated with C5aR [22]. The C5aR-mediated pathway may be involved in neurodegenerative lesions of AD, and abnormal activation of this signaling pathway can lead to cell apoptosis, leading to neurodegenerative diseases [23, 24]. These studies all indicate the relationship between neuronal C5a and AD progression. However, there are few reports on the changes in serum C5a levels in patients with AD of different severity and its correlation with inflammatory response and cognitive function. This study investigated the changes in serum C5a levels in patients with AD of different severity, and analyzed their relationship with inflammatory factors and cognitive function, so as to provide references for clinical prevention and treatment in AD.

## Materials and methods

### Ethics statement

The experiments were authorized by the academic ethics committee of the First People´s Hospital of Jingzhou City. All procedures were strictly implemented according to *Declaration of Helsinki*. All subjects involved were fully informed of the study objective and signed the informed consent before sampling.

### Study subjects

AD patients diagnosed in the First People’s Hospital of Jingzhou City from July 2019 to June 2021 were treated to improve their cognitive function and enhance their behavioral and life abilities, and 240 AD patients who met the requirements were selected as the study subjects, including 107 males and 133 females, aged 58–85 years. All participants underwent clinical interviews and related neurocognitive assessments, as well as necessary laboratory or imaging examinations. According to the clinical dementia rating scale (CDR) score, AD patients were assigned to 3 subgroups: mild group (CDR score 1, 78 cases), moderate group (CDR score 2, 85 cases), and severe group (CDR score 3, 77 cases). Another 80 healthy subjects who underwent physical examinations in the First People’s Hospital of Jingzhou City during the same period were selected as the control group, including 37 males and 43 females, aged 58–85 years. AD was diagnosed based on the core clinical standards of The National Institute on Aging and the Alzheimer’s Association [25]. The CDR score was strictly executed according to the standards, and was operated by trained physicians. There was a semi-structured interview between the operator and the patients, where the physician asked the patient 6 questions, scored them based on the their response, and asked the informed or caregivers to understand the patient’s daily living conditions.

### Inclusion and exclusion criteria

Inclusion criteria were as follows: (1) meeting the diagnostic criteria of AD; (2) with decreased cognitive function, executive function, and motor control; (3) with complete Mini-Mental State Examination (MMSE) data.

Exclusion criteria were as follows: (1) with infectious or inflammatory diseases; (2) with immune system diseases; (3) with brain diseases such as intracranial space occupying, brain trauma, epilepsy, or cerebrovascular diseases; (4) with Lewy body dementia, vascular dementia, and other cognitive impairment [with the aid of positron emission tomography (PET) imaging, Lewy body dementia was diagnosed by the reduction of dopamine transporter (DAT) uptake; vascular dementia was diagnosed according to the DSM-IV diagnostic criteria, and could also be examined through CT or MRI imaging]; (5) with severe heart, liver and kidney failure; (6) with drug-induced, toxic, hypothyroidism, and other secondary dementia; (7) with anxiety, depression, schizophrenia, and other mental diseases; (8) with malignant tumor disease.

### Data and sample collection

The following clinical baseline data of selected subjects were collected: age, sex (male/female), body mass index (BMI), disease duration, education level, smoking history, drinking history, diabetes, hypertension, systolic blood pressure (SBP), diastolic blood pressure (DBP), and MMSE data. Serum total cholesterol (TC), triglycerides (TG), low-density lipoprotein (LDL), and high-density lipoprotein (HDL) levels were determined using a fully automatic biochemical analyzer in strict accordance with the product instructions, by the same batch of personnel from the laboratory on the same instrument, with each sample repeated 3 times. MMSE can assess the cognitive function of patients, including 5 tasks: registration (3 scores), orientation (10 scores), recall (3 scores), attention and calculation (5 scores), and language (9 scores), with a total score of 30. A lower total score represented worse cognitive function [26]. On the next morning, 10 mL of fasting venous blood of each enrolled patient after admission was collected into an ordinary blood collection tubes without anticoagulant, and 6 mL of venous blood was collected, allowed to stand for 30 min, and centrifuged for 10 min at 4 °C at 3000 r/min, and the supernatant was collected for subpackage in eppendorf (EP) tubes, labeled, and stored at a refrigerator at -80 °C. The rest 4 mL of venous blood was placed in the ethylenediaminetetraacetic acid anticoagulant tubes, allowed to stand, centrifuged, and the plasma was collected for subpackage in EP tubes, labeled, and stored at -80 °C.

#### Enzyme-linked immunosorbent assay (ELISA)

The serum C5a level and serum inflammatory factors including tumor necrosis factor-α (TNF-α), interleukin (IL)-1β, IL-6, C-reactive protein (CRP) levels, and plasma Aβ40, Aβ42 levels were assessed using the ELISA kit (Amylet Scientific, Wuhan, Hubei, China), and the Aβ42/Aβ40 value was calculated. The operations were conducted according to kit instructions.

### Statistical analysis

GraphPad Prism 8 (GraphPad Software Inc., San Diego, CA, USA) and SPSS 21.0 (IBM Corp. Armonk, NY, USA) were adopted for data analysis and mapping. Shapiro-Wilk test was employed for normal distribution test. Data were expressed as mean ± standard deviation, with non-paired *t* test applied for comparisons between 2 groups, and one-way analysis of variance (ANOVA) applied for comparisons among groups, followed by Tukey’s multiple comparisons test. Fisher’s exact test was adopted for comparative analysis of categorical variables. Logistic multivariate regression analysis was employed to evaluate the independent risk factors for AD exacerbation. The diagnostic value of serum C5a level in AD patients was analyzed using the receiver operating characteristic (ROC) curve. Kaplan-Meier curve was used to analyze the effect of serum C5a level on the cumulative incidence of severe AD. *P* < 0.05 was indicative of statistical significance.

## Results

### Comparisons of general clinical data of AD patients with different severity

A total of 320 subjects were included in this study, including 80 healthy subjects (Non-AD group), 78 patients with mild AD, 85 patients with moderate AD, and 77 patients with severe AD. Comparative analysis of clinical data of mild, moderate and severe AD groups and Non-AD group manifested that there were no significant differences among the 4 groups in sex, BMI, disease duration, educational level, smoking and drinking history, diabetes, hypertension, SBP, DBP, TC, TG, LDL, and HDL levels (all *P* > 0.05), while there were statistical differences between mild AD and Non-AD groups, and among the mild, moderate and severe groups in age (all *P* < 0.05). In addition, there was statistical difference in Aβ42/Aβ40 ratio between severe AD and mild AD groups, and among moderate, severe groups and Non-AD group (all *P* < 0.05) (Table 1).

**Table 1:** Comparisons of general clinical data of AD patients of different severity

Characteristics	Non-AD(N = 80)	Mild AD(N = 78)	Moderate AD (N = 85)	Severe AD(N = 77)	*P*_a_	*P*_b_	*P*_c_	*P*_d_	*P*_e_	*P*_f_
Age (year)	75.36 ± 9.32	69.36 ± 5.24	74.54 ± 6.16	76.76 ± 6.25	< 0.001	< 0.001	0.046	< 0.001	0.504	0.273
Sex (male/female)	37/43	35/43	41/44	31/46	0.754	0.627	0.344	0.875	0.876	0.52
BMI (kg/m^2^)	26.17 ± 2.64	25.67 ± 3.06	26.28 ± 2.67	26.32 ± 2.72	0.176	0.164	0.925	0.273	0.791	0.726
Disease duration (year)	*/*	3.56 ± 1.42	3.64 ± 1.53	3.78 ± 1.60	0.731	0.367	0.57	/	/	/
Education level (year)		7.60 ± 3.02	6.72 ± 2.64	6.78 ± 2.54	0.103	0.152	0.989		0.215	0.276
	7.24 ± 2.72							0.432		
Smoking (yes/no)	31/49	34/44	31/54	30/47	0.424	0.626	0.749	0.628	0.872	0.999
Drinking (yes/no)	36/44	32/46	42/43	36/41	0.345	0.519	0.755	0.633	0.641	0.873
Diabetes (yes/no)	33/47	29/49	41/44	30/47	0.205	0.869	0.269	0.628	0.434	0.871
Hypertension (yes/no)	35/45	26/52	32/53	24/53	0.625	0.864	0.412	0.194	0.433	0.138
SBP (mm/Hg)	129.78 ± 12.92	132.24 ± 12.51	128.92 ± 12.41	133.67 ± 15.26	0.257	0.785	0.065	0.226	0.663	0.086
DBP (mm/Hg)	78.91 ± 9.15	78.45 ± 9.36	80.62 ± 10.24	81.48 ± 9.57	0.332	0.131	0.841	0.755	0.261	0.087
TC (mmol/L)	4.27 ± 1.91	4.29 ± 1.87	4.34 ± 1.93	4.37 ± 2.02	0.985	0.964	0.994	0.947	0.815	0.75
TG (mmol/L)	1.72 ± 0.86	1.64 ± 0.78	1.76 ± 0.82	1.81 ± 0.93	0.637	0.423	0.925	0.541	0.76	0.53
LDL (mmol/L)	2.81 ± 0.63	2.78 ± 0.64	2.91 ± 0.57	2.89 ± 0.62	0.364	0.501	0.976	0.767	0.286	0.424
HDL (mmol/L)	1.18 ± 0.34	1.20 ± 0.31	1.16 ± 0.28	1.22 ± 0.34	0.689	0.915	0.437	0.7	0.68	0.462
Aβ42/Aβ40 ratio	0.27 ± 0.06	0.29 ± 0.07	0.31 ± 0.09	0.32 ± 0.09	0.118	0.022	0.481	0.055	0.001	< 0.001

Note: BMI: body mass index; SBP: systolic blood pressure; DBP: diastolic blood pressure; TC: total cholesterol; TG: triglyceride; LDL: low density lipoprotein; HDL: high density lipoprotein; Aβ40: amyloid β-protein 40; Aβ42: amyloid β-protein 42. The measurement data were expressed by mean ± standard deviation or number of cases. Fisher’s exact test was employed for the comparative analysis of categorical variables. One-way ANOVA was applied for data comparisons among groups of continuous variables, followed by Tukey’s multiple comparisons test was used for the post test. *P* < 0.05 was indicative of statistical significance. *P*_a_: the moderate group compared with the mild group; *P*_b_: the severe group compared with the mild group; *P*_c_: the severe group compared with the moderate group; *P*_d_: the mild group compared with the Non-AD group; *P*_e_: the moderate group compared with the Non-AD group; *P*_f_: the severe group compared with the Non-AD group

### Comparison of serum C5a, pro-inflammatory factors and MMSE scores in AD patients with different severity and healthy subjects

Serum C5a and pro-inflammatory factor (TNF-α, IL-1β, IL-6, CRP) levels in AD patients with different severity and Non-AD group were determined by ELISA. Serum C5a, TNF-α, IL-1β, IL-6 and CRP levels in AD group was higher than those of the Non-AD group (all *P* < 0.05). Among them, patients with severe AD manifested elevated C5a level relative to the mild and moderate groups (all *P* < 0.05), while no significant difference between the moderate AD group and the mild AD group (*P* = 0.122). TNF-α, IL-1β, IL-6, and CRP levels between the severe AD group and the mild and moderate AD groups were statistically significant (all *P* < 0.05), while TNF-α, IL-1β, IL-6, and CRP levels between the mild AD group and the moderate AD group were not significantly different (*P* = 0.055, 0.067, 0.093, 0.088). Additionally, the MMSE score of the AD group was lower than those of the Non-AD group (all *P* < 0.05). There was a statistically significant difference in MMSE score between the severe AD group and the mild and moderate AD groups and also between the mild and moderate AD groups (all *P *< 0.05) (Table 2).

**Table 2:** Comparisons of serum C5a, pro-inflammatory factors, and MMSE scores between AD patients of different severity and healthy subjects

Indexes		Non-AD (N = 80)		Mild AD (N = 78)		Moderate AD (N = 85)		Severe AD (N = 77)		*P*_a_			*P*_b_			*P*_c_			*P*_d_		*P*_e_		*P*_f_
C5a (pg/mL)	64.28 ± 9.18		68.29 ± 9.14		70.56 ± 9.45		78.97 ± 9.26		0.122			< 0.0001			< 0.0001			0.007		< 0.0001		< 0.0001	
TNF-α (pg/mL)	182.35 ± 13.41		187.36 ± 16.48		192.52 ± 17.44		202.75 ± 19.86		0.055		< 0.0001			0.0006			0.038			< 0.0001		< 0.0001	
IL-1β (pg/mL)	24.37 ± 4.91		32.47 ± 5.82		34.29 ± 6.68		39.62 ± 7.14		0.067		< 0.0001			< 0.0001			< 0.0001			< 0.0001		< 0.0001	
IL-6 (pg/mL)	61.37 ± 9.72		89.58 ± 12.41		93.34 ± 15.67		103.75 ± 16.21		0.093		< 0.0001			< 0.0001			< 0.0001			< 0.0001		< 0.0001	
CRP (pg/mL)	1.84 ± 0.37		3.98 ± 0.41		4.11 ± 0.54		4.62 ± 0.63		0.088		< 0.0001			< 0.0001			< 0.0001			< 0.0001		< 0.0001	
MMSE scores (points)	29.32 ± 3.84		14.75 ± 0.58		14.42 ± 0.77		11.09 ± 1.02		0.026		< 0.0001			< 0.0001			< 0.0001			< 0.0001		< 0.0001	

Note: C5a: Complement factor 5a; TNF-α: tumor necrosis factor-α; IL-1β: interleukin (IL)-1β; IL-6: interleukin (IL)-6; CRP: C-reactive protein; MMSE: Mini-Mental State Examination. The measurement data were expressed by mean ± standard deviation. One-way ANOVA was applied for data comparisons among groups of continuous variables, followed by Tukey’s multiple comparisons test was used for the post test. *P* < 0.05 was indicative of statistical significance. *P*_a_: the moderate group compared with the mild group; *P*_b_: the severe group compared with the mild group; *P*_c_: the severe group compared with the moderate group; *P*_d_: the mild group compared with the Non-AD group; *P*_e_: the moderate group compared with the Non-AD group; *P*_f_: the severe group compared with the Non-AD group

### Correlation analysis of serum C5a level with pro-inflammatory factors, MMSE scores and Aβ42/Aβ40 ratio in AD patients

**Fig. 1:**
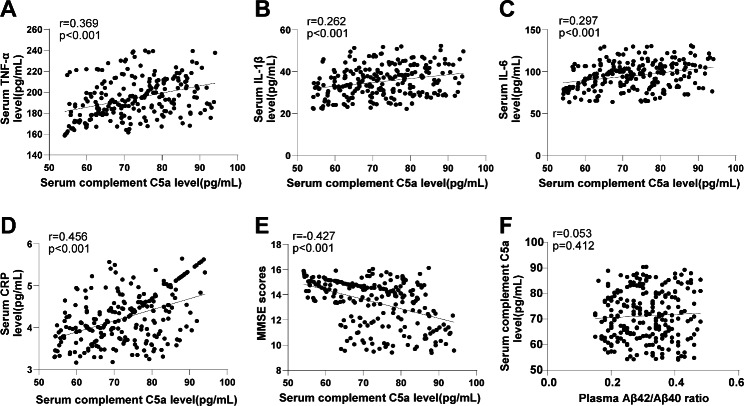
Correlation analysis of serum C5a level with pro-inflammatory factors, MMSE scores and Aβ42/Aβ40 ratio. (**A**-**E**) The correlations between serum C5a level and pro-inflammatory factors (TNF-α, IL-1β, IL-6, CRP) and the MMSE score were analyzed by Pearson test. (**F**) Correlation analysis between plasma Aβ42/Aβ40 ratio and serum C5a level. Data in panels **A**-**F** were analyzed by Pearson coefficient

To further explore the relationship between serum C5a level, inflammatory factor level, and cognitive function in AD patients, the correlation of serum C5a level with inflammatory factor (TNF-α, IL-1β, IL-6, CRP) and MMSE score was analyzed by Pearson test. Serum C5a level was positively correlated with TNF-α, IL-1β, IL-6, and CRP levels (r = 0.369, 0.262, 0.297, 0.456) (all *P* < 0.001) (Fig. 1A-D), and was negatively correlated with the MMSE score (r = -0.427) (*P* < 0.001) (Fig. 1E). In addition, there was no significant correlation between plasma Aβ42/Aβ40 ratio and serum C5a level (r = 0.053) (*P* > 0.05) (Fig. 1F). The results indicated that serum C5a level was not affected by Aβ accumulation, and was closely associated with serum pro-inflammatory factors, which could reflect the inflammatory state of the body to a certain extent, and could reflect the cognitive function of AD patients.

### Serum C5a level is one of the independent risk factors for the exacerbation in AD patients

To further explore the relationship between serum C5a level and the severity of AD patients, AD severity was taken as the dependent variable, and age, TNF-α, IL-1β, IL-6, CRP, and serum C5a levels were included in the binary logistic regression analysis model combining with the previous analysis results. After adjusting age, IL-1β and CRP levels, serum C5a level was on of the independent risk factors for AD aggravation (P = 0.001, OR = 1.070, 95%CI: 1.027–1.115) (Table 3).

**Table 3:** Logistic multivariate regression analysis of risk factors for the aggravation of AD patients

Variable	*P* value	OR	OR95%CI
Age	0.003	1.088	1.030–1.150
TNF-α	0.352	1.010	0.989–1.031
IL-1β	0.003	1.087	1.028–1.150
IL-6	0.068	1.024	0.998–1.050
CRP	0.017	2.245	1.158–4.353
C5a	0.001	1.070	1.027–1.115

Note: TNF-α: tumor necrosis factor-α; IL-1β: interleukin (IL)-1β; IL-6: interleukin (IL)-6; CRP: C-reactive protein; C5a: Complement factor 5a; OR: Odds Ratio; CI: Confidence interval

### The occurrence of severe AD might be related to a stimulated serum C5a level

**Fig. 2:**
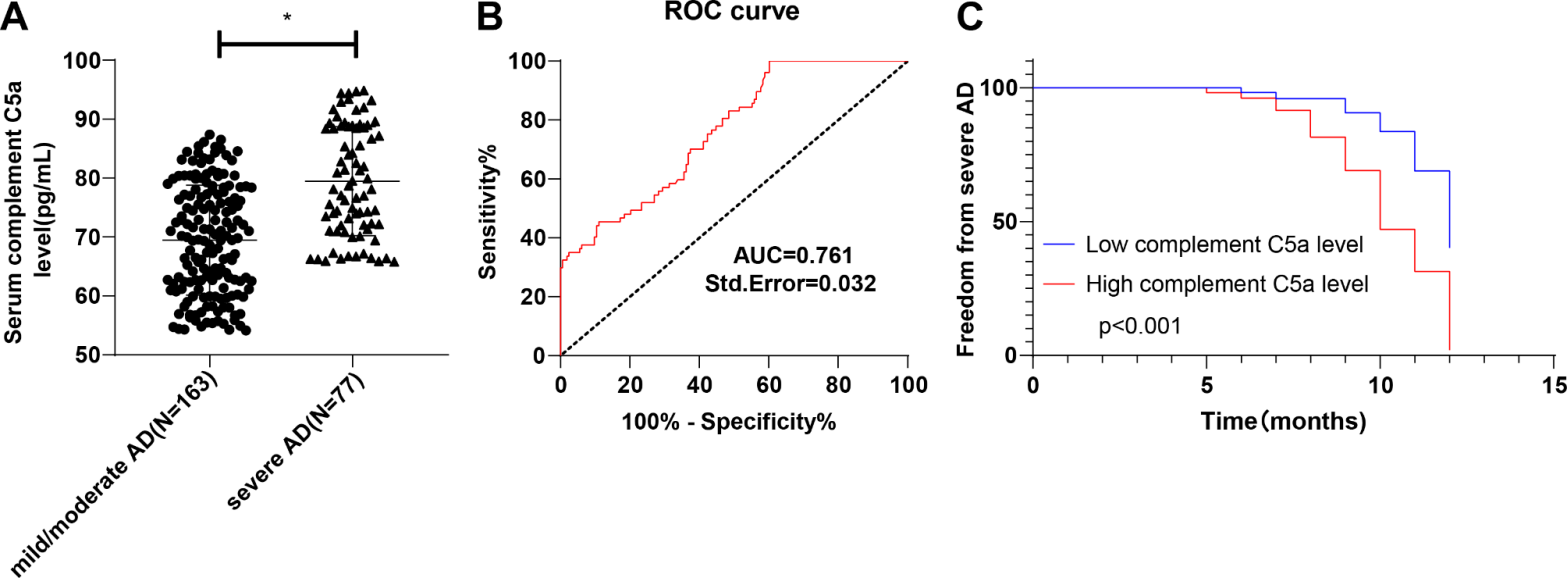
The difference of serum C5a level between mild, moderate and severe AD patients. (**A**) Serum C5a level was determined by ELISA; (**B**) The ROC curve of serum C5a level for severe AD evaluation; (**C**) Kaplan-Meier curve was employed to analyze the cumulative incidence of severe AD. Data in panel **A** were tested by independent *t* test. Data in panel **B** were analyzed using the ROC curve. * *P* < 0.05

Firstly, serum C5a level in severe, moderate and mild AD groups was compared (*P* < 0.05) (Fig. 2A). ROC curve of serum C5a level for AD severity assessment was plotted (Fig. 2B). The area under the curve (AUC) was 0.761, and the cut-off value was 65.78 pg/mL (sensitivity 100.00%, specificity 39.88%), indicating that serum C5a level > 65.78 pg/mL assisted in the diagnosis of severe AD patients. In addition, AD patients were allocated to the low C5a expression group (C5a < 65.78pg/mL, N = 72) and high C5a expression group (C5a ≥ 65.78 pg/mL, N = 168) based on the cut-off value of ROC curve analysis of serum C5a level. The incidence of severe AD was compared between the 2 groups. There were differences in the incidence of severe AD between the 2 groups (χ^2^ = 7.540, *P* < 0.05). The incidence of severe AD in the high C5a expression group was 37.50% (63/168), higher than that in the low C5a expression group (14/72, 19.44%) (Table 4). Moreover, Kaplan-Meier curve analysis manifested that the curve of the C5a overexpression group shifted to the left (*P* < 0.05, Fig. 2C), indicating that the cumulative incidence of severe AD in the C5a overexpression group was higher under the same evaluation cycle. The results indicated that the occurrence of severe AD might be associated with a facilitated serum C5a level.

**Table 4:** Prognosis of high and low serum C5a expression groups

		Severe AD		Mild to moderate AD	Total
C5a low expression group	14		58		72
C5a high expression group	63		105		168
Total	77		163		240

## Discussion

AD is the most prevalent cause of dementia, which is a growing global health problem with huge implications for both society and individuals [2]. Evidence has shown that C5a can interfere with neuropathologic and neuroinflammation alterations in AD mouse models [27]. This study investigated the changes in serum C5a levels in AD patients with different severity and found that serum C5a level was augmented with the severity of AD, and its expression was positively correlated with serum pro-inflammatory factor levels, and negatively correlated with cognitive function.

An existing study has reported that the peripheral immune system is believed to affect the pathology of the central nervous system in AD, and the peripheral adaptive immune response, especially T cell-mediated peripheral adaptive immune response, may play a role in the pathogenesis of AD [28]. In addition, blood biomarkers have high specificity and sensitivity, which, as newly developed biomarkers, can identify various pathological processes, including neuroinflammation, synaptic dysfunction, metabolic damage, protein aggregation, and neurodegeneration [29]. Therefore, non-invasive and easily obtainable peripheral inflammatory biomarkers should be considered as potential biomarkers for early diagnosis of AD. The complement system, as an important component of the innate immune system, plays an important role in the inflammatory damage of AD nerve after activation. Therefore, measurement of serum C5a levels can reflect the condition of AD disease and has important clinical significance. Accumulating data support that the essential AD CSF biomarker Aβ42 reflects key elements of the pathophysiology of AD [6]. Our results revealed that mild AD patients and non-AD subjects, and mild, moderate and severe AD patients manifested statistical differences in age and Aβ42/Aβ40 ratio. Consistently, disease-linked mutations in presenilin genes cause stimulated production Aβ42, which is the predominant form found in AD [30]. The complement system is a main component of inflammatory responses, which may have a crucial property in neurodegenerative conditions including AD [31]. Our results manifested that the serum levels of C5a, TNF-α, IL-1β, IL-6 and CRP in AD patients were higher than those of the non-AD subjects, with the levels in severe AD patients facilitated compared with those of mild and moderate AD patients, while there was no significant differences in these levels between mild and moderate AD patients. Likewise, C5a is a potent effector of microglial movement, which is up-regulated in a variety of neurodegenerative diseases, including AD [32, 33]. C5a-C5aR1 signaling largely exerts its functions in AD by promoting microglial activation pathways, which accelerate the progression of the disease [17]. However, there is a little report on serum C5a levels in AD patients of different severity. This study manifested for the first time that C5a level was intensified with the severity of AD. Inflammation exists in the pathologically vulnerable regions of AD brains, which is related to the full complexity of the local peripheral inflammatory responses [34]. Serum inflammation status is evaluated by TNF-α, IL-1β, IL-6, and CRP [35]. Our results elicited that severe AD patients had statistical differences in TNF-α, IL-1β, IL-6, and CRP levels with mild and moderate AD patients. Similarly, pro-inflammatory cytokine TNF-α is related to agitation severity in AD [36] and IL-1β is implicated in contributing to disease severity in various central nervous system diseases including AD [37]. MMSE score is the most commonly used scale to assess cognitive impairment [38]. Our findings elicited that AD patients demonstrated lowered MMSE scores relative to the non-AD subjects, with statistical difference in MMSE scores between severe and moderate AD patients, and no statistical difference between mild and moderate AD patients.

Complement activation in the central nervous system is a double-edged sword. Brain complement activation may promote tissue damage by inducing neuronal cell death and promoting the release of pro-inflammatory cytokines through activated glial cells. On the other hand, complement activation can have beneficial effects on the brain by promoting neuroprotection and tissue remodeling [16]. Complement system activation plays an important role in AD neuroinflammatory injury. Complements in serum are generally secreted by macrophages, neutrophils, natural killer cell, dendritic cells and other immune cells. Complements in the central nervous system are mainly produced by activation of microglia [31, 39]. Complement C5a is produced in the process of complement activation, which can strongly attract neutrophils, astrocyte and microglia in the brain to gather in the area of complement activation, and has a strong function of promoting inflammation [40]. Therefore, determination of serum C5a level can reflect the inflammatory state of the body to a certain extent. Repressing the activation of the complement system can reduce the induction of inflammatory factors by diminishing the level of C5a, thus reducing the release of inflammatory factors, which has certain value in preventing and treating AD. Therefore, assessing serum C5a level has certain clinical significance in AD. Our research showed that serum C5a level was the independent risk factors for worsening AD, and high C5a level was the risk factor for severe AD. Subsequently, we analyzed correlations of serum level of C5a with pro-inflammatory factors, MMSE score, and Aβ42/Aβ40 ratio and found a positive correlation between serum level of C5a and the levels of TNF-α, IL-1β, IL-6, and CRP, and a negative correlation between serum level of C5a and MMSE score. However, previous study has shown that complement can be activated by Aβ [41]. Importantly, C5a can also synergistically exert damaging effects with Aβ, causing neurological deficits [42].To exclude the possibility that high level of serum C5a might be considered as a result of a accumulation, we found no significant correlation between plasma Aβ42/Aβ40 ratio and serum C5a level through Pearson correlation analysis (correlation coefficient r = 0.053, *P* > 0.05), which could prove that high level of C5a in the blood was not the result of Aβ accumulation. Similarly, activation of complement proteins mediates the expression of chemokines and pro-inflammatory cytokines (such as IL-1 and TNF-α) [43]. Complement protein induces cognitive decline and neuroinflammation after traumatic brain injury at extended chronic time points [44]. Collectively, serum level of C5a was not influenced by plasma Aβ42/Aβ40 ratio, and was closely related to serum pro-inflammatory factors, which reflected the inflammatory state and cognitive function of AD patients. Furthermore, through logistic regression analysis, we discovered that after age, IL-1β, and CRP level adjustment, serum level of C5a was one of the independent risk factors for AD aggravation. Similarly, complement receptor 1 gene variation is identified in recent studies as a risk factor for AD [45]. To conclude, augmented serum levels of C5a promoted the risk of AD exacerbation. Moreover, we plotted ROC curves and found that serum level of C5a > 65.78 pg/mL assisted in severe AD diagnosis and discovered for the first time that the occurrence of severe AD might be associated with an amplified serum C5a level.

In summary, our findings supported that serum C5a level was one of the independent risk factors for AD exacerbation, which pave the way for the clinical treatment of AD. The activation of the complement system plays a crucial role in the neuroinflammatory injury of AD. However, we only preliminarily discussed the correlations between C5a level change and inflammatory response and cognitive function in AD patients with different severity but did not deeply explore the role of C5a level in the pathogenesis of AD. Moreover, the specific relationship between C5a and cognitive function of AD patients was not studied in depth. Additionally, the number of cases and events included in this study for analysis is relatively small, and further expansion of sample size and multicenter studies are needed to further clarify the clinical significance of serum C5a level in AD. Furthermore, due to the limitations in experimental period and funding, we are currently unable to obtain a sufficient sample size for other dementia patients (non AD patients) to verify the results of serum C5a level. Furthermore, measuring peripheral blood indicators may not necessarily reflect the internal situation of the brain, and serum C5a may be influenced by various factors such as coagulation processes, which needs further study to explore the correlation between severe AD and serum C5a levels in multiple aspects. These deficiencies need to be supplemented in future studies to clarify the relationship between C5a and inflammation from the molecular and cellular levels.

## Data Availability

All the data generated or analyzed during this study are included in this published article.

## Abbreviations

AD: Alzheimer’s disease
C5a: Complement factor 5a
MMSE: Mini-Mental state examination
ROC: Receiver operating characteristic
Aβ: Amyloid β
CDR: Clinical dementia rating scale
BMI: Body mass index
SBP: Systolic blood pressure
DBP: Diastolic blood pressure
TC: Total cholesterol
TG: Triglycerides
LDL: Low-density lipoprotein
HDL: High-density lipoprotein
ELISA: Enzyme-linked immunosorbent assay
TNF-α: Tumor necrosis factor-α
IL: Interleukin
CRP: C-reactive protein
ANOVA: Analysis of variance
