# Correction: Correlation of serum complement factor 5a level with inflammatory response and cognitive function in patients with Alzheimer’s disease of different severity

**DOI:** 10.1186/s12883-023-03410-4

**Published:** 2023-10-02

**Authors:** Zhilian Li, Huifang Wu, Yi Luo, Xianpei Tan

**Affiliations:** 1 Department of Neurology, The First People’s Hospital of Jingzhou City, No.8 HangKong Road, Shashi District, 434100 Jingzhou City, Hubei Province P.R. China; 2 Yangtze University, 434023 Jingzhou City, Hubei Province P.R. China

**Correction: BMC Neurol 23, 319 (2023)**

10.1186/s12883-023-03256-w

Following publication of the original article [1], the authors reported an error in the affiliations. Authors Zhilian Li, Yi Luo and Xianpei Tan should be affiliated to “The First People’s Hospital of Jingzhou City” and author Huifang Wu should be affiliated to “Yangtze University”.

The original article [1] has been corrected.
